# Pre-expulsion of the Placenta in Child-birth

**Published:** 1856-05

**Authors:** C. Goodbrake

**Affiliations:** Clinton, Illinois


ARTICLE II.—*Pre-expulsion of the Placenta in Child-birth.* By C. GOODBRAKE, of Clinton, Ill., March, 1856.

I was sent for in great haste to visit Mrs. B., residing 1½ miles out of town, on the 21st of February, at 9 o'clock, A.M., and who, I was informed by the messenger (her father,) was in the eighth month of her second pregnancy, and had that morning slipped on the ice before the door of the house, and fell with her back across a hewn stick of timber about eight inches square. On arriving at the house, I found the woman lying across the foot of the bed on her left side; her hands and feet cold, pulse feeble but quick, and complaining of a dull heavy pain in the lumber region, over which her mother informed me there was considerable abrasion of the skin, caused by the stick of timber, over which she had fallen. Upon inquiry she informed me, there was *very moderate* hemorrhage, and that she had only experienced two or three *slight* bearing-down pains since receiving the fall, but complained of a continuous pain in her back, and an uneasy sensation in her right side, and said something had torn loose in her side when she fell.

The sensation in her side, she described as a gurgling of water, and said it caused her to feel sick. In addition to these symptoms there appeared to be considerable nervous excitement.

After considering the nature of the case, and the symptoms as they presented themselves to my mind, I came to the conclusion, that by giving such remedies as would quiet pain, allay nervous excitement, and check the hemorrhage, the case might possiby be safely conducted to the full period of gestation. With this view, I prescribed a powder composed of morphine, camphor, and acetate of lead; ordered the woman to be placed properly in the bed; directed warmth to be applied to her hands and feet, and enjoined strict quiet.

I now stepped into an adjoining room to the fire; but the changing of the woman's position in bed, brought on violent pains with increased flooding, and her mother soon called me to the bed-side, and informed me that the water had come away. I immediately proceeded to make an examination, and to my surprise found the placenta already protruding through the external parts, and before I had time to make any further examination, there came on a violent pain and the placenta came away entire; the head of the child following the placenta so far as to press against the perineum.

I immediately placed my finger in the mouth of the child, and while in this position I distinctly felt the convulsive or gasping movements of the child, and in a few seconds the woman had another violent and protracted pain which expelled the child. When the child was born it did not breathe, but seemed to be affected with a general tremor, every muscle perfectly rigid, with arms and legs flexed, face bloated, and lips purple. I immediately instituted artificial respiration, when to my great satisfaction, and the unbounded joy of the relatives of the little premature, it first began to breathe on its own responsibility and then to squall. After the birth of the child, the uterus could be felt above the pubes, firm and well contracted, and there was no more hemorrhage. Mother and child (female sex) are both doing well up to this time, March 10th.

I must not neglect to state, that about three months previous to the accident above detailed, this same woman received a fall by the upsetting of an omnibus, at which time she was considerably bruised and a good deal frightened. And she informed me that ever since that time she had experienced more or less of the "uneasy sensation" in her side, and which was only aggravated by her fall on the ice.

So far as I can now recollect, I have never read of a case similar to the one I have attempted to describe. I have been unfortunate enough to have several cases of Placenta Previa come under my care, consequently I have endeavored to neglect no opportunity, by reading or otherwise, to inform myself on the subject, and to my mind, at least, this case presents the following points of interest:—

*First.* The very slight degree of hemorrhage for a case of *reversed birth*, there being no more blood lost than in the majority of cases of ordinary labor. This might be taken as an argument in favor of the plan recommended by some writers in cases of Placenta Previa, namely, the detachment of the placenta to avoid hemorrhage.

*Second.* The child being born alive after the expulsion of the placenta. How was life sustained from the time the placenta became detached and expelled, until respiration was established?

